# Alterations in Functional Network Topology Within Normal Hemispheres Contralateral to Anterior Circulation Steno-Occlusive Disease: A Resting-State BOLD Study

**DOI:** 10.3389/fneur.2022.780896

**Published:** 2022-03-22

**Authors:** Junjie Wu, Fadi Nahab, Jason W. Allen, Ranliang Hu, Seena Dehkharghani, Deqiang Qiu

**Affiliations:** ^1^Department of Radiology and Imaging Sciences, Emory University School of Medicine, Atlanta, GA, United States; ^2^Department of Neurology, Emory University School of Medicine, Atlanta, GA, United States; ^3^Department of Pediatrics, Emory University School of Medicine, Atlanta, GA, United States; ^4^Joint Department of Biomedical Engineering, Emory University and Georgia Institute of Technology, Atlanta, GA, United States; ^5^Department of Radiology, New York University Langone Medical Center, New York, NY, United States; ^6^Department of Neurology, New York University Langone Medical Center, New York, NY, United States

**Keywords:** cerebrovascular reserve, functional magnetic resonance imaging, graph theory, functional connectivity, cerebrovascular disease

## Abstract

The purpose of this study was to assess spatially remote effects of hemodynamic impairment on functional network topology contralateral to unilateral anterior circulation steno-occlusive disease (SOD) using resting-state blood oxygen level-dependent (BOLD) imaging, and to investigate the relationships between network connectivity and cerebrovascular reactivity (CVR), a measure of hemodynamic stress. Twenty patients with unilateral, chronic anterior circulation SOD and 20 age-matched healthy controls underwent resting-state BOLD imaging. Five-minute standardized baseline BOLD acquisition was followed by acetazolamide infusion to measure CVR. The BOLD baseline was used to analyze network connectivity contralateral to the diseased hemispheres of SOD patients. Compared to healthy controls, reduced network degree (*z*-score = −1.158 ± 1.217, *P* < 0.001, false discovery rate (FDR) corrected), local efficiency (*z*-score = −1.213 ± 1.120, *P* < 0.001, FDR corrected), global efficiency (*z*-score = −1.346 ± 1.119, *P* < 0.001, FDR corrected), and enhanced modularity (*z*-score = 1.000 ± 1.205, *P* = 0.002, FDR corrected) were observed in the contralateral, normal hemispheres of SOD patients. Network degree (*P* = 0.089, FDR corrected; *P* = 0.027, uncorrected) and nodal efficiency (*P* = 0.089, FDR corrected; *P* = 0.045, uncorrected) showed a trend toward a positive association with CVR. The results indicate remote abnormalities in functional connectivity contralateral to the diseased hemispheres in patients with unilateral SOD, despite the absence of macrovascular disease or demonstrable hemodynamic impairment. The clinical impact of remote functional disruptions requires dedicated investigation but may portend far reaching consequence for even putatively unilateral cerebrovascular disease.

## Introduction

Patients with chronic steno-occlusive disease (SOD) of the cerebrovascular system are at risk of ischemic stroke (1, 2). Cerebral hypoperfusion and impaired hemodynamics, which can be evaluated by cerebrovascular reactivity (CVR), lead to ischemic or potentially selective neuronal damage among these patients (3). Previous resting-state fMRI studies in stroke and cerebrovascular disease have detected blood oxygen level-dependent (BOLD) connectivity disruptions within several brain networks, including motor, attention, frontoparietal and default mode networks (4–8), and may include long range changes distant or even contralateral to areas of injury, such as in motor networks or interhemispheric functional connectivity (9, 10).

Observations of remote and widespread network dysfunction (11) have compelled investigation into its relation to hemodynamic failure and autoregulation. Graph theory provides an effective tool for capturing the topological principles that govern brain networks (12, 13). Under this framework, numerous lines of evidence support that brain organization is shaped by an optimal balance between functional segregation and integration (14, 15), as well as a trade-off between wiring costs, principles of minimum work, and efficiency of parallel information transfer (16). This configuration can be influenced by brain maturation (17), cognitive states (18–20), and brain diseases (21, 22). While disrupted organization of functional networks has been found in stroke and cerebrovascular disease (23–26), little is known about whether network topology in normal-appearing brain areas contralateral to lesions is similarly vulnerable.

A challenge to such paradigms, however, rests in the nature of BOLD functional network connectivity, which is influenced both by changes in neurovascular coupling and true neural connections (27, 28). In cerebrovascular disease, neurovascular coupling is likely impaired, particularly in the diseased hemispheres, which can undermine attempts to recover BOLD changes and thus faithful representations of connectivity (29, 30). Importantly, however, as even normal-appearing brain parenchyma contralateral to SOD may be affected by sub-clinical hemodynamic compromise and increased stroke risk (31, 32), we anticipated that remote, occult alterations in the hemodynamic response function could accompany or even drive changes in BOLD functional connectivity within the putatively normal hemispheres of such patients. Examining BOLD networks in the contralateral hemispheres and their associations with CVR may deepen our understanding of functional brain networks in cerebrovascular disease and potentially illuminate well-recognized, non-ischemic consequence of SOD such as cognitive and other functional impairments (33).

In the present study, we aimed to investigate remote effects of hemodynamic compromise on BOLD functional connectivity of the contralateral hemispheres in unilateral, chronic cerebral SOD, hypothesizing the presence of measurable disturbances in functional connectivity remote and even contralateral to SOD in patients with unilateral macrovascular disease on cross-sectional or catheter angiographic cerebrovascular imaging. Graph-theory metrics of functional networks were estimated using resting-state BOLD, addressing two questions: (i) As compared to healthy controls, do observable network alterations exist within the contralateral, normal hemispheres in SOD patients? (ii) Does network connectivity correlate with cerebral hemodynamics as measured by CVR in the normal-appearing hemispheres of the patients?

## Materials and Methods

### Participants

Twenty patients with unilateral chronic SOD of the anterior circulation were studied (age, 49.00 ± 11.38 years; range, 29–70 years; seven males, 13 females; seven patients with right-sided disease), including eight patients with occlusion or high-grade (>75%) stenosis of the cervical internal carotid artery (ICA), 6 patients with occlusion or high-grade (>75%) stenosis of the intracranial ICA, 1 patient with occlusion of the middle cerebral artery, and five patients with idiopathic intracranial SOD. For each patient, the diseased hemisphere was determined on 3D time-of-flight MR angiography (*N* = 20), as well as on CT angiography (*N* = 7) or digital subtraction angiography (*N* = 13) when available. All imaging was assessed by a vascular neurologist (F.N.) and one of neuroradiologists (J.W.A. and S.D.), each with >10 years of experience.

Twenty age-matched healthy controls (age, 40.75 ± 16.21 years; range, 20-68 years; 12 males, 8 females) were enrolled for comparison. This study was approved by the Institutional Review Board at Emory University School of Medicine. Informed consent was obtained from healthy controls and retrospective review of patient data was performed.

### Data Acquisition and Preprocessing

MR imaging was performed on a Siemens Tim Trio 3-Tesla scanner (Siemens Healthcare, Erlangen, Germany) equipped with a 32-channel head array coil in all patients with SOD. Twenty-minute (600 volumes) continuous BOLD data were collected using a gradient-echo echo-planar imaging (EPI) sequence (TR/TE = 2,000/30 ms, FA = 78°, FOV = 220 × 220 mm^2^, matrix = 64 × 64, slice thickness = 4 mm, 30 slices). After 5 min of baseline BOLD acquisition, acetazolamide (ACZ) (1 gram dissolved in 10 mL normal saline) was slowly infused intravenously over 3–5 min, followed by normal saline flush. The healthy controls underwent resting-state BOLD imaging without ACZ enhanced BOLD measurement for 13.33 min (400 volumes) using the same scan protocol with the same MRI scanner and coil models (Siemens Tim Trio with 32-channel head array coil). To avoid bias, the same duration of BOLD measurement from both patients and healthy controls was used for network analysis, as detailed below. In both patient and control groups, a high-resolution 3D anatomical image was also acquired using an axial T_1_-weighted magnetization-prepared rapid acquisition with gradient echo (MPRAGE) sequence (TR/TE = 1,900/3.52 ms, FA = 9°, FOV = 216 × 256 mm^2^, matrix = 216 × 256, slice thickness = 1 mm, 176 slices).

Images were processed using SPM8 (Wellcome Trust Center for Neuroimaging, University College London, London, UK) and MATLAB (MathWorks, Natick, Massachusetts, USA). Anatomical MPRAGE image was co-registered to BOLD images, and segmented to create gray matter, white matter (WM) and cerebrospinal fluid (CSF) masks. BOLD data preprocessing comprised removal of the first 10 volumes (20 s), slice-timing correction, motion correction, normalization to Montreal Neurological Institute space, and spatial smoothing with a 6-mm Gaussian kernel. For network analysis, the first 5 min of data were used, and underwent linear detrending, regression of nuisance signals and temporal band-pass filtering (0.01–0.1 Hz). The nuisance signals included head-motion profiles as well as WM and CSF signals. WM and CSF signals were extracted from contralateral hemispheres in the patients to eliminate contamination from lesions (8), and from both left and right hemispheres in the healthy controls.

### CVR Calculation

CVR was measured by estimating the cerebrovascular response to ACZ. Although breath holding or inhalation of gas with increased CO_2_ concentration could be also used for CVR measurement, both approaches have their disadvantages. Breath-holding approach requires good subject cooperation and is difficult to control CO_2_ concentration in the lungs. While inhalation of gas with increased CO_2_ concentration is used in studies and has its advantages, complex gas delivery and sampling apparatus are required, limiting its translation to clinical settings. In contrast, ACZ is a safe and highly tolerated agent, and its intravenous administration is a relatively easy and accepted medical procedure in most patients (34).

In the patients, the mean BOLD images before (BOLD_pre−ACZ_) and after (BOLD_post−ACZ_) ACZ administration were generated by averaging the first and the last 30 volumes (1 min, not including previously discarded volumes) of BOLD data, respectively. CVR maps were then determined as CVR = (BOLD_post−ACZ_ – BOLD_pre−ACZ_) / BOLD_pre−ACZ_ × 100% (34).

### Network Construction and Analysis

A network (i.e., graph) comprising *nodes* and *edges* that connect the nodes was established separately for each hemisphere for the purposes of hemispheric analysis. For the patients, the normal-appearing hemispheres contralateral to SOD were analyzed. For the healthy controls, individual hemispheres were analyzed separately to produce hemispheric, reference standard connectivity profiles for comparison and normalization. Nodes were defined by regions of interest (ROIs) in Anatomical Automatic Labeling atlas (35) that divides each hemisphere into 45 regions, and edges represented interregional functional connectivity, measured by Pearson correlation coefficients. Symmetric correlation matrices were built after computing the Pearson correlation coefficients of the average temporal signals between all pairs of ROIs. To convert the correlation matrices to sparse networks, a sparsity threshold, defined as the ratio of the number of actual edges to the maximum possible number of edges in a network, was used. The sparsity-based thresholding method ensures the same number of edges in the resultant networks by applying a network-specific correlation threshold. Currently, there is no definitive way to accurately determine thresholds, therefore the sparsity thresholds in this study were set to 20, 30, and 40%, which are within typical sparsity ranges of human neuron networks (36, 37).

Graph theory was applied to quantify the topological properties of weighted undirected networks using Brain Connectivity Toolbox (38) (http://www.brain-connectivity-toolbox.net). We calculated node-based network metrics including node degree, nodal efficiency *E*_*nodal*_, local efficiency *E*_*local*_ and betweenness centrality, and global network metrics including global efficiency *E*_*global*_, modularity and assortativity coefficient. Degree is the sum of all edge weights connected to a node. Nodal efficiency *E*_*nodal*_ is defined as the reciprocal of the harmonic mean of shortest path lengths between a given node and all other nodes. The average *E*_*nodal*_ across all nodes can be used to evaluate global efficiency *E*_*global*_ of parallel information processing in a network. Local efficiency *E*_*local*_ is the global efficiency calculated in node neighborhoods. *E*_*local*_ is an indicator of fault tolerance, measuring how well the information is transferred between the immediate neighbors of a given node when it is eliminated. Betweenness centrality is determined as the fraction of all shortest paths in the network that pass through a given node, thereby used to detect important nodes for information transfer. Modularity is defined as the ability of a network to be decomposed into subnetworks that are more linked within modules than between modules. Given that the optimal modular structure may be slightly different from run to run owing to heuristics in the algorithm (39), the modularity analysis was repeated 500 times and the averaged modularity was calculated. Assortativity coefficient is a correlation coefficient for the degrees of neighboring nodes, which measures how often nodes with a certain degree are connected to nodes with a similar degree. As an indicator of resilience, assortativity coefficient reflects network vulnerability to insult.

### Statistical Analysis

**Figure 1:**
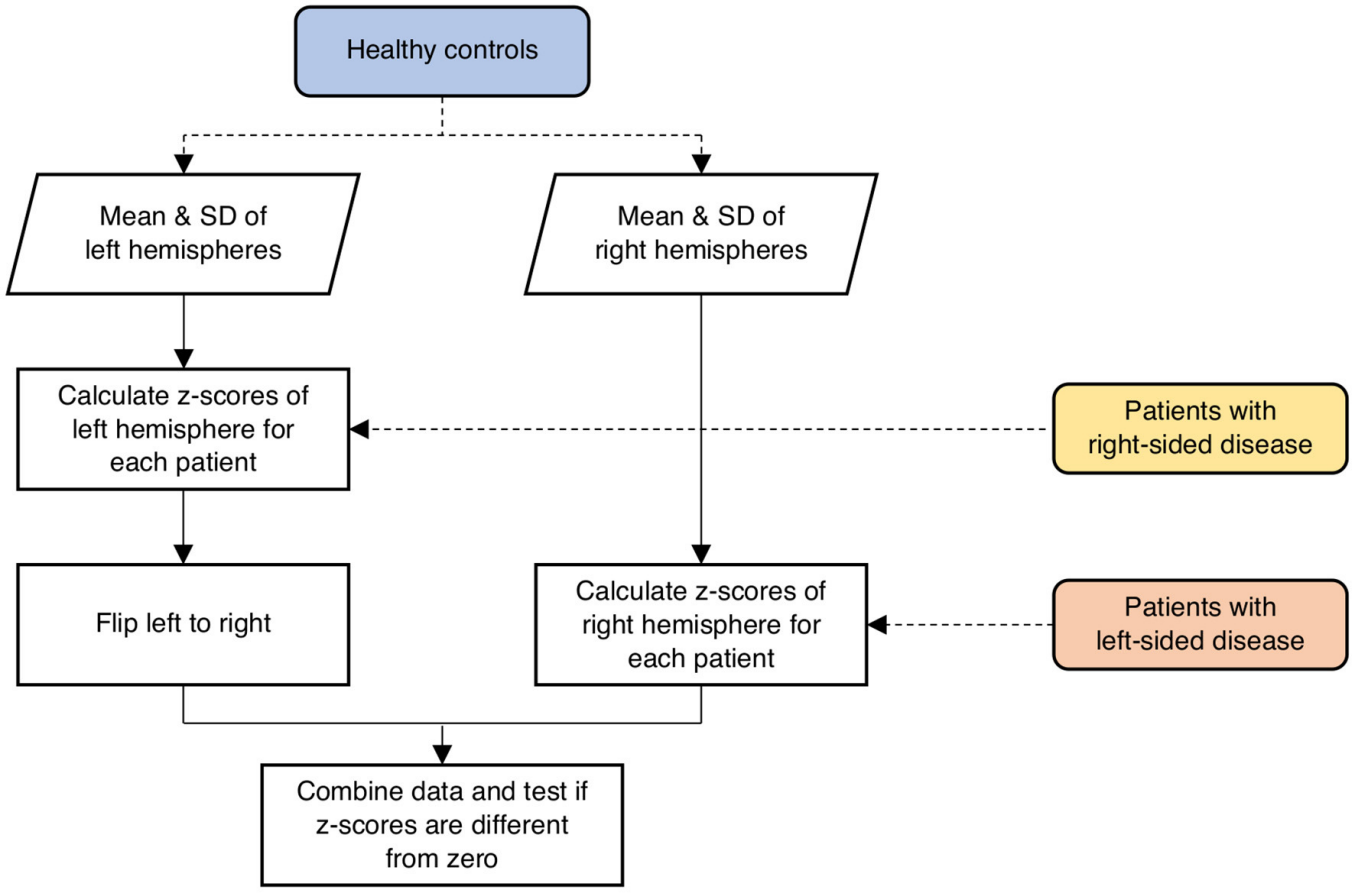
Schematic illustration of the procedure involved in calculating *z*-scores of network parameters in the contralateral hemispheres of the patients relative to the respective hemispheres of the healthy controls. SD, standard deviation.

In the healthy controls, the mean and standard deviation (SD) of network measures were calculated for both left and right hemispheres separately as a reference standard for the patients. To evaluate abnormalities in the hemispheres contralateral to SOD in the patient population, network parameters were scored in terms of *z*-values relative to the mean and SD of the respective hemispheres across all the healthy controls calculated above (Figure 1). Specifically, if a patient had right-sided disease, *z*-scores of network parameters in the left hemisphere were calculated relative to the mean and SD of the left hemispheres across all controls. One-sample *t*-test was performed to test if the *z*-scores had a mean of zero. The correlations between network parameters and ROI-specific CVR were evaluated using a linear mixed-effects model, with subject-specific slopes and intercepts modeled as random effects to assess the linear relationships between test variables. The false discovery rate (FDR; *Q* < 0.05) method was employed to correct for multiple comparisons.

## Results

**Figure 2:**
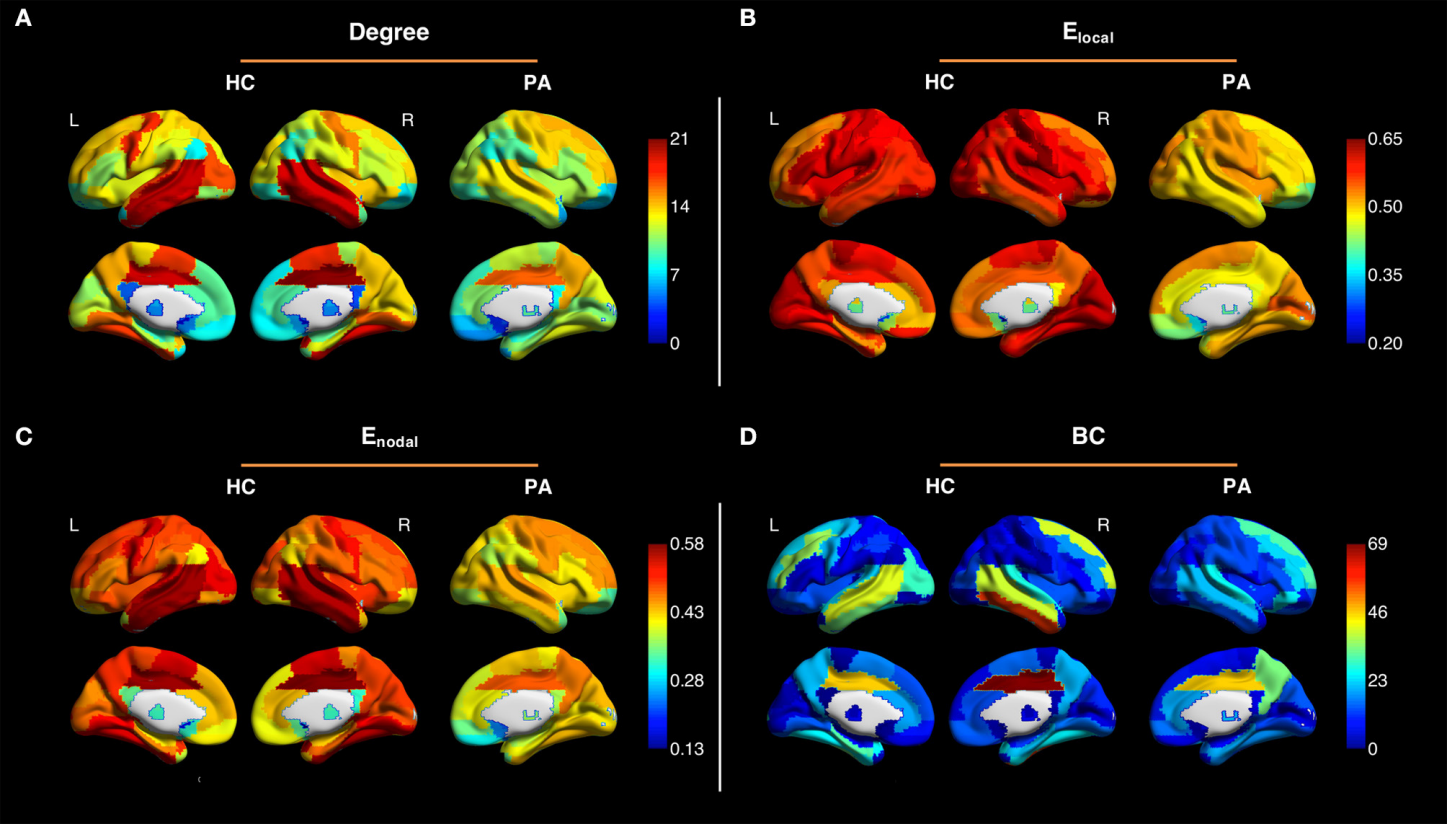
Averaged maps of degree **(A)**, local efficiency *E*_*local*_ **(B)**, nodal efficiency *E*_*nodal*_ **(C)**, and betweenness centrality (BC) **(D)** in the left and right hemispheres of the healthy controls (HC) and in the contralateral hemispheres of the patients (PA) (connectivity sparsity = 40%). The maps of the patients with right-sided disease were left-right flipped to create the averaged maps. Overall, degree, *E*_*local*_ and *E*_*nodal*_ were lower in the patients as compared to the healthy controls.

**Figure 3:**
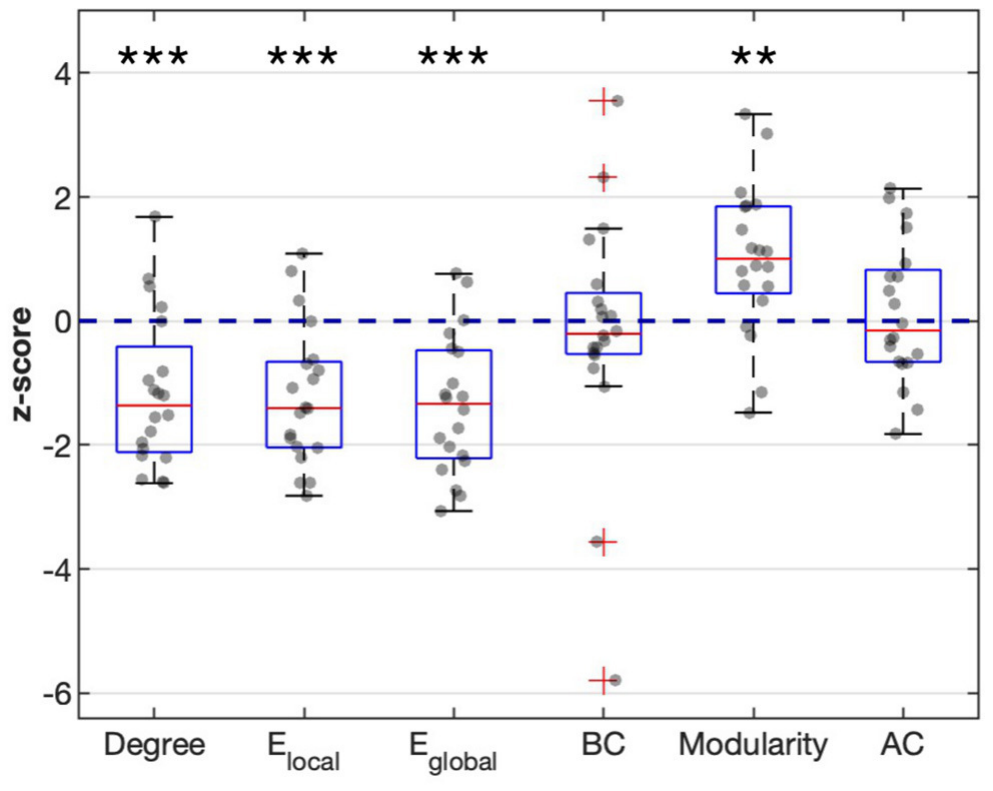
Z-scores of degree, local efficiency *E*_*local*_, global efficiency *E*_*global*_, betweenness centrality (BC), modularity, and assortativity coefficient (AC) in the contralateral hemispheres of the patients relative to the respective hemispheres of the healthy controls (connectivity sparsity = 40%). The z-scores of degree, *E*_*local*_, and *E*_*nodal*_ were significantly lower than zero, while the *z*-score of modularity was significantly higher than zero. Asterisks indicate statistical significance determined by one-sample *t*-test (^**^P < 0.005, ^***^P < 0.001; false discovery rate corrected).

Figure 2 shows the averaged maps of degree, local efficiency *E*_*local*_, nodal efficiency *E*_*nodal*_, and betweenness centrality in both hemispheres of the healthy controls and in the contralateral hemispheres of the patients. In general, degree, *E*_*local*_ and *E*_*nodal*_ were lower in the patients as compared to the healthy controls. These observations were confirmed by *z*-scores of network parameters in the contralateral hemispheres of the patients relative to the respective hemispheres of the controls (Figure 3). One-sample *t*-test showed that at connectivity sparsity = 40%, the z-scores of degree (*P* < 0.001, FDR corrected), *E*_*local*_ (*P* < 0.001, FDR corrected), and *E*_*gobal*_ (*P* < 0.001, FDR corrected) were lower than zero, suggesting compromised network strength and efficiency in the patient cohort. The z-score of modularity was higher than zero (*P* = 0.002, FDR corrected), which suggests that the brain networks are subdivided into more clearly delineated groups in the patients. Similar results were obtained at connectivity sparsity = 20 and 30% (Table 1).

**Table 1:** Mean *z*-scores of network parameters in the contralateral hemispheres.

	**Connectivity sparsity = 20%**		**Connectivity sparsity = 30%**		**Connectivity sparsity = 40%**	
	***z*-score (mean ±SD)**	***P*-value^**a**^**	***z*-score (mean ±SD)**	***P*-value^**a**^**	***z*-score (mean ±SD)**	***P*-value^**a**^**
Degree	−1.108 ± 1.131	0.002	−1.146 ± 1.186	<0.001	−1.158 ± 1.217	<0.001
*E_*local*_*	−0.868 ± 1.191	0.006	−1.409 ± 1.272	<0.001	−1.213 ± 1.120	<0.001
*E_*global*_*	−0.835 ± 1.022	0.005	−1.306 ± 1.056	<0.001	−1.346 ± 1.119	<0.001
Betweenness Centrality	0.284 ± 0.933	0.226	−0.038 ± 1.453	0.907	−0.195 ± 1.931	0.656
Modularity	0.721 ± 0.920	0.005	0.786 ± 1.115	0.008	1.000 ± 1.205	0.002
Assortativity Coefficient	−0.111 ± 1.091	0.654	0.065 ± 1.163	0.907	0.127 ± 1.130	0.656

a *P-value of one-sample t-test with false discovery rate correction to assess for zero mean z-scores*.

*SD, standard deviation; E_local_, local efficiency; E_global_, global efficiency*.

**Figure 4:**
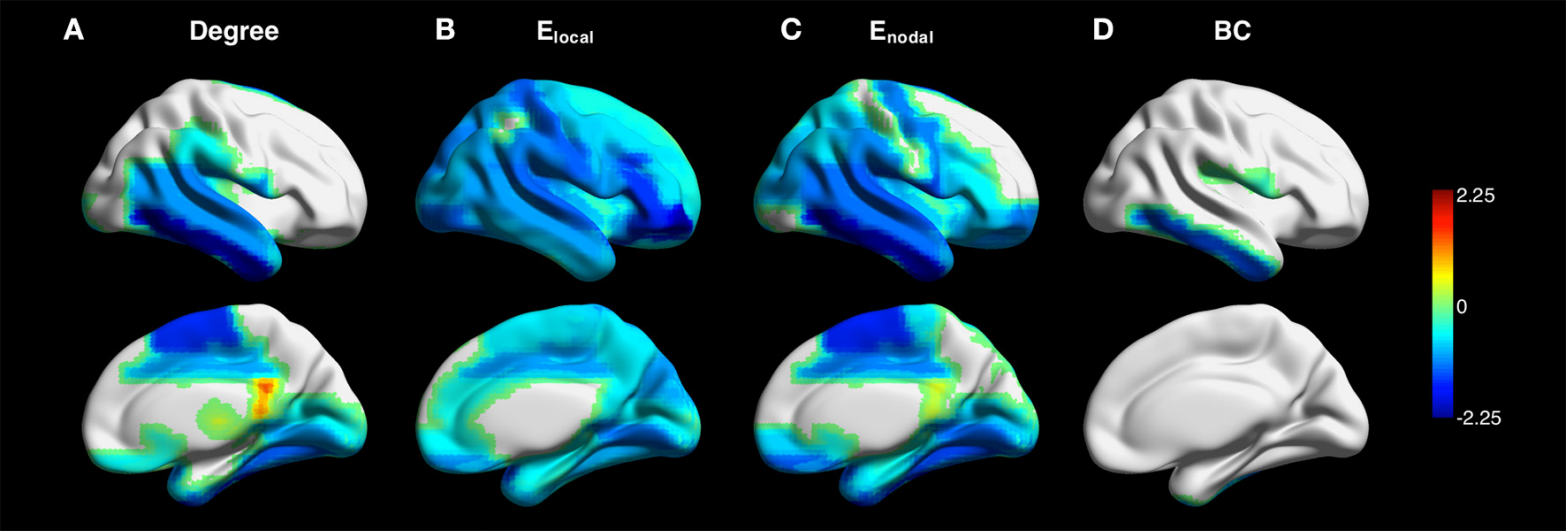
*Z*-maps of degree **(A)**, local efficiency *E*_*local*_ **(B)**, nodal efficiency *E*_*nodal*_ **(C)**, and betweenness centrality (BC) **(D)** in the contralateral hemispheres averaged across all patients (connectivity sparsity = 40%). The maps of the patients with right-sided disease were left-right flipped to create the averaged maps. One-sample *t*-test with false discovery rate correction showed that negative *z*-scores of degree were primarily observed in the supplementary motor, fusiform and temporal cortices. Negative z-scores were found across the brain for *E*_*local*_ and *E*_*nodal*_. The *z*-scores of betweenness centrality were negative in the Rolandic operculum and inferior temporal gyrus.

Figure 4 shows the z-maps of degree, *E*_*local*_, *E*_*nodal*_, and betweenness centrality in the contralateral hemispheres averaged over all patients. One-sample *t*-test with FDR correction showed that negative *z*-scores of degree were primarily observed in the supplementary motor, fusiform and temporal cortices. The *z*-scores of network efficiency were lower than zero across the brain. The *z*-scores of betweenness centrality were negative in the Rolandic operculum and inferior temporal gyrus (see Supplementary Table 1 for more details).

**Figure 5:**
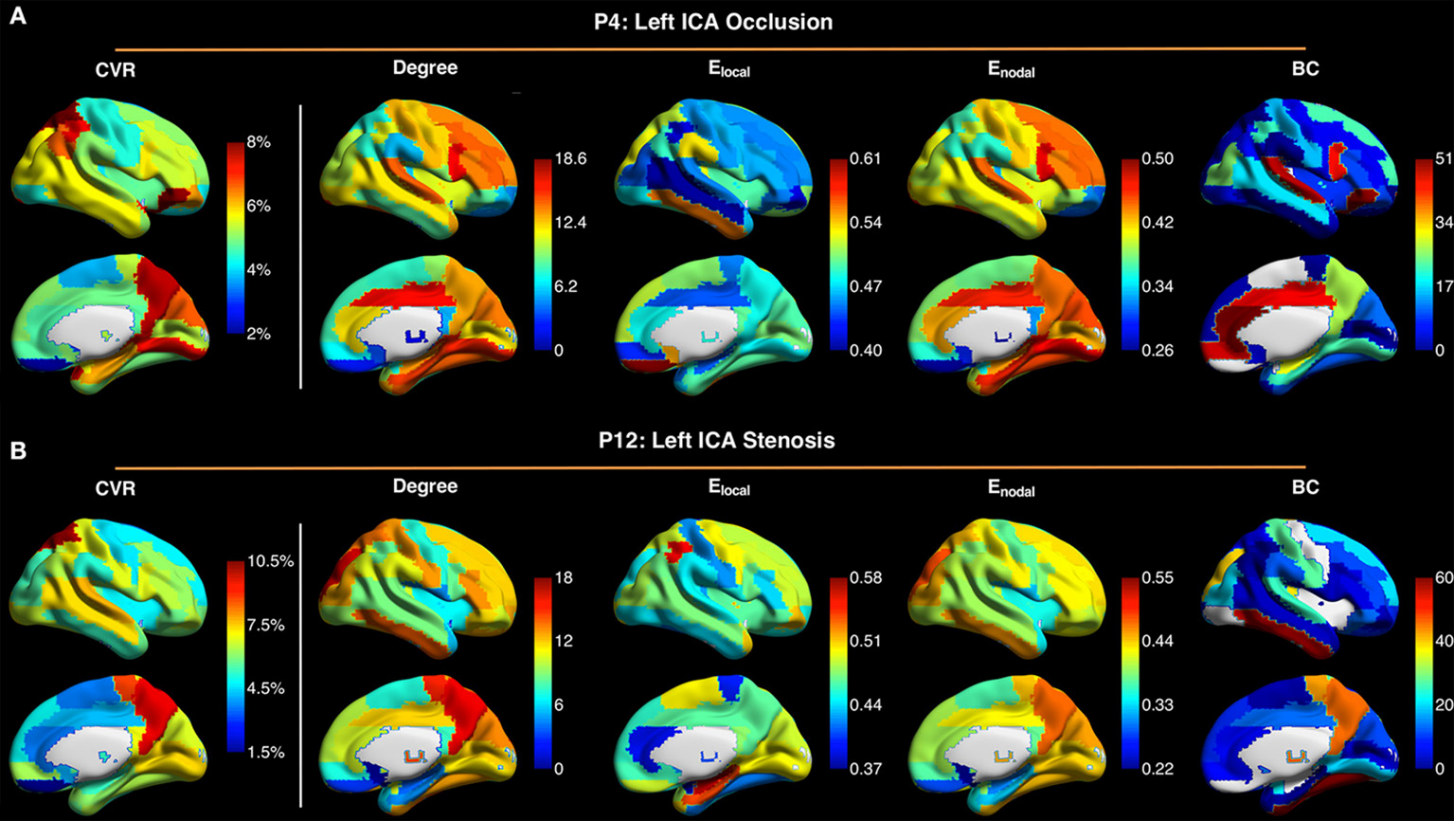
Maps of cerebrovascular reactivity (CVR), degree, local efficiency *E*_*local*_, nodal efficiency *E*_*nodal*_, and betweenness centrality (BC) in the contralateral hemispheres in a patient with occlusion of left internal carotid artery (ICA) (P4) **(A)**, and in a patient with stenosis of the left ICA (P12) **(B)**. The CVR maps are similar to the maps of degree and *E*_*nodal*_. Connectivity sparsity was set to 40%.

**Figure 6:**
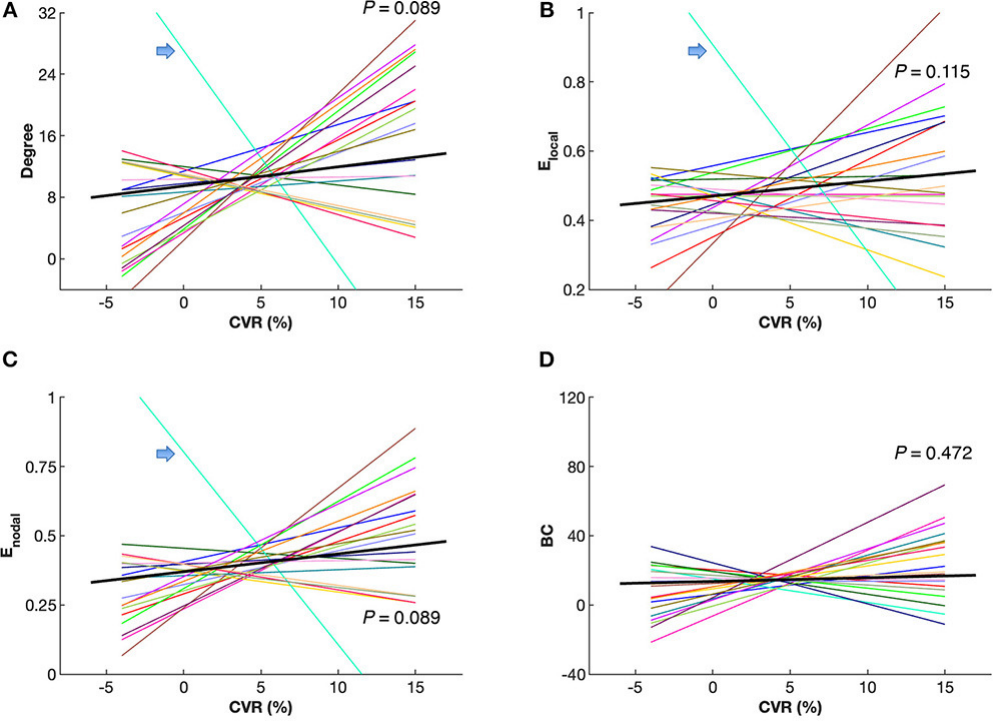
Correlations between cerebrovascular reactivity (CVR) and network parameters, including degree **(A)**, local efficiency *E*_*local*_ **(B)**, nodal efficiency *E*_*nodal*_ **(C)** and betweenness centrality (BC) **(D)** in the contralateral hemispheres of the patients. Mixed-effects model showed a trend toward significant correlations between CVR and degree, as well as between CVR and *E*_*nodal*_ (*P* < 0.10, false discovery rate corrected). Each colored line represents an individual patient. The black bold lines indicate overall trends across patients. Arrows indicate a single patient (P9) with a trend deviating from the others. Connectivity sparsity was set to 40%.

Figure 5 shows maps of CVR and network parameters in the contralateral hemispheres of two representative patients. Visual inspection suggested relationships between degree and CVR, as well as between *E*_*nodal*_ and CVR. Mixed-effects model showed a trend toward significant correlations between degree and CVR (*P* = 0.089, FDR corrected; *P* = 0.027, uncorrected), and between *E*_*nodal*_ and CVR (*P* = 0.089, FDR corrected; *P* = 0.045, uncorrected), while significance was not observed between *E*_*local*_ and CVR (*P* = 0.115, FDR corrected; *P* = 0.086, uncorrected), and between betweenness centrality and CVR (*P* = 0.472, FDR corrected; *P* = 0.472, uncorrected) (Figure 6). For the correlations of degree, *E*_*local*_ and *E*_*nodal*_ with CVR, one patient exhibited a trend deviating from the remainder of the cohort (arrows in Figure 6). To estimate the influences of the potential outlier, the same analyses were repeated after excluding this participant. After the outlier exclusion, the associations between degree and CVR (*P* = 0.023, FDR corrected), and between *E*_*nodal*_ and CVR (*P* = 0.023, FDR corrected) became significant.

## Discussion

This study reveals altered organization of BOLD functional brain networks contralateral to unilateral anterior circulation steno-occlusive disease. Reduced degree and efficiency, and enhanced modularity of functional networks were observed in normal-appearing hemispheres contralateral to SOD in patients as compared to healthy controls, indicating potentially long-range consequence to hemodynamic impairment manifesting in network disruptions or reorganization even remote from areas of apparent disease. The network degree and nodal efficiency furthermore showed a close relationship with CVR, suggesting that cerebral hemodynamics may be predictive of BOLD network metrics.

fMRI has been widely utilized to explore brain function (40) including in cerebrovascular disease and stroke (41–43). Compared to task-related fMRI, resting-state fMRI does not involve explicit or intentional task performance. Flexible *post-hoc* analyses for estimating intrinsic functional connectivity can be applied on resting-state data to explore multiple functional networks, thus resting-state imaging is potentially well-suited to clinical settings, including patients unable to perform complex tasks.

While most fMRI studies have interpreted BOLD signal as a measure of neural activity, based in the assumption that neurovascular coupling is relatively consistent (27), neurovascular coupling may be disrupted in patients with cerebrovascular disease, especially in affected hemispheres (29, 30). Moreover, based on comparisons with healthy controls, previous studies (31, 32) have reported compromised CVR even in putatively normal contralateral hemispheres, suggesting that a BOLD hemodynamic response function could be altered even contralateral to regions of macrovascular disease and without demonstrable vascular pathology on cross-sectional or catheter angiographic imaging.

BOLD functional networks likely reflect underlying physiology. Recent fMRI studies in healthy participants have reported relationships between network efficiency and cerebral blood flow (CBF) (44), and between functional connectivity and CVR (45). In this work, we further explored the physiological basis of functional connectivity among a patient group with cerebrovascular disease. Our results showed a trend toward significant correlations between either network degree or nodal efficiency and vascular reserve as quantified by CVR, implying that cerebral hemodynamics may at least partially account for the observed changes in functional networks. Importantly, the direction of influence between CVR and functional connectivity was not studied directly, and the findings do not specifically implicate the presence of intrinsic vascular pathology in the contralateral hemisphere, wherein a remote functional influence upon local vasoreactivity could alternatively modulate augmentation profiles. This potential impact of neurovascular uncoupling and perhaps anatomic variables including the extent of vascular connectivity (for instance across the anterior communicating artery) also cannot be easily disambiguated from our results; nevertheless, the correlations suggest that caution is merited when assessing BOLD functional connectivity among patients with cerebrovascular compromise, and that corrections for CVR effects should be contemplated when studying even remote BOLD connectivity in cerebrovascular disease.

Cerebrovascular disease may produce not only territorial, lacunar, and borderzone ischemia, but also demonstrable silent infarctions or even subclinical damage below the resolution of conventional imaging. It should be noted that neurophysiological changes occur even in anatomically intact areas (46). Remote alterations have been observed in neuronal activity, cortical metabolism and CBF (9). To further investigate remote manifestations, we examined the organization of apparent functional networks contralateral to cerebrovascular compromise. The results showed that network strength and efficiency were impaired in normal-appearing hemispheres, while the brain networks were subdivided into more clearly delineated groups. The most compromised brain regions were observed in the temporal lobe. Our findings corroborate the notion that apparent network dysfunction extends into the structurally intact regions (9, 25). However, as noted above it remains unclear to what extent such alterations in the contralateral hemispheres result from true differences in neuronal functional connectivity versus changes in neurovascular coupling. Systematic investigations are necessary to evaluate neurovascular coupling further within areas free of macrovascular disease.

Several issues remain to be addressed. First, the information of handedness and education was not collected in the participants. A balance between these factors is needed in the future. Second, a trend toward significant correlations of network degree and nodal efficiency with CVR was observed after FDR correction, perhaps due to the limited statistical power associated with the relatively small sample size in the present study. Future studies with larger sample size are needed to confirm this finding. Third, we investigated network parameters in the network level, where network analysis was performed for each hemisphere. For the analysis in module level, the statistical power is limited owing to the relatively small sample size in this study. Further studies with larger sample size are needed for the analysis in module level. Fourth, relatively large ROIs were selected to construct networks in the current study. Assessing functional connectivity strength to investigate voxel-wise changes in the patients is a focus of future study in our group. Fifth, single-band single-echo EPI with relatively low temporal (TR = 2 s) and spatial (voxel size = 3.4 × 3.4 × 4 mm^3^) resolution was employed for BOLD imaging in this study. A recently developed multi-band multi-echo EPI (47) can be used to increase temporal and/or spatial resolution (48, 49) and functional contrast-to-noise (50), thereby improving measurement accuracy. Finally, with an assumption of temporal stationarity, functional connectivity was calculated across the whole scan duration, and the future study of resting-state functional networks in terms of temporal fluctuations among patients with stroke and cerebrovascular disease may offer further mechanistic insights into the nature of remote functional connectivity in such patients.

In conclusion, our study provides evidence for altered BOLD functional connectivity contralateral to the diseased hemispheres in patients with unilateral SOD. The close relationship between network connectivity and CVR further suggests that caution should be taken when evaluating BOLD brain networks in cerebrovascular disease, even in regions with putatively normal macrovasculature.

## Data Availability Statement

The raw data supporting the conclusions of this article will be made available by the authors, without undue reservation.

## Ethics Statement

The studies involving human participants were reviewed and approved by Institutional Review Board at Emory University School of Medicine. The patients/participants provided their written informed consent to participate in this study.

## Author Contributions

JW, FN, SD, and DQ contributed to study concept and design. JW, FN, JA, RH, SD, and DQ contributed to data acquisition and analysis and contributed to drafting the manuscript and figures. All authors contributed to the article and approved the submitted version.

## Funding

This research was supported by internal fund of Emory University and National Institutes of Health (R01AG072603).

## Conflict of Interest

SD — UNRELATED: Unpaid scientific consultation and collaboration with Ischemaview; Past travel support from Ischemaview as an individual; Scientific consulting with Regeneron as an individual; Domestic and international patent filed by institution for inventor's disclosure in the development of device to measure brain electrical properties; Grant funding paid to institution from undisclosed donor for study on the development of remote stroke detection devices. The remaining authors declare that the research was conducted in the absence of any commercial or financial relationships that could be construed as a potential conflict of interest.

## Publisher's Note

All claims expressed in this article are solely those of the authors and do not necessarily represent those of their affiliated organizations, or those of the publisher, the editors and the reviewers. Any product that may be evaluated in this article, or claim that may be made by its manufacturer, is not guaranteed or endorsed by the publisher.
