# Supplementary material for: Alterations in Functional Network Topology Within Normal Hemispheres Contralateral to Anterior Circulation Steno-Occlusive Disease: A Resting-State BOLD Study
Source: Front Neurol. 2022 Mar 22;13:780896. doi: 10.3389/fneur.2022.780896 (PMC8980268; doi:10.3389/fneur.2022.780896)
Supplement: Supplementary file 1 [file Table_1.DOCX]

***Supplementary Material***

**Supplementary Table 1.** Regional z-scores of network parameters in the contralateral hemispheres. Regions of interest with non-zero mean z-scores (one-sample *t*-test, false discovery rate corrected) are listed.

| Region | z-score | Region | z-score |
| --- | --- | --- | --- |
| **Degree** |  | ***E_local_*** |  |
| Rolandic operculum | -1.06 | Precental gyrus | -0.75 |
| Supplementary motor area | -1.50 | Superior frontal gyrus, dorsolateral | -0.47 |
| Olfactory cortex | -0.54 | Superior frontal gyrus, orbital part | -0.59 |
| Gyrus rectus | -0.57 | Middle frontal gyrus | -0.66 |
| Median cingulate and paracingulate gyri | -0.95 | Middle frontal gyrus, orbital part | -2.00 |
| Posterior cingulate gyrus | 1.29 | Inferior frontal gyrus, opercular part | -0.97 |
| Calcarine fissure and surrounding cortex | -0.55 | Inferior frontal gyrus, triangular part | -1.45 |
| Lingual gyrus | -0.97 | Inferior frontal gyrus, orbital part | -1.51 |
| Fusiform gyrus | -1.28 | Rolandic operculum | -1.05 |
| Supramarginal gyrus | -0.55 | Supplementary motor area | -0.63 |
| Thalamus | 0.67 | Superior frontal gyrus, medial orbital | -0.57 |
| Heschl gyrus | -0.58 | Gyrus rectus | -1.04 |
| Superior temporal gyrus | -1.11 | Insula | -0.97 |
| Temporal pole: superior temporal gyrus | -1.12 | Anterior cingulate and paracingulate gyri | -0.51 |
| Middle temporal gyrus | -1.02 | Median cingulate and paracingulate gyri | -1.00 |
| Temporal pole: middle temporal gyrus | -0.61 | Hippocampus | -0.62 |
| Inferior temporal gyrus | -2.01 | Parahippocampal gyrus | -0.47 |
|  |  | Amygdala | -0.60 |
| ***E_nodal_*** |  | Calcarine fissure and surrounding cortex | -0.60 |
| Precental gyrus | -1.11 | Cuneus | -0.99 |
| Superior frontal gyrus, orbital part | -1.12 | Lingual gyrus | -1.16 |
| Middle frontal gyrus, orbital part | -1.12 | Superior occipital gyrus | -1.13 |
| Inferior frontal gyrus, opercular part | -0.81 | Middle occipital gyrus | -0.79 |
| Inferior frontal gyrus, triangular part | -0.59 | Inferior occipital gyrus | -1.25 |
| Inferior frontal gyrus, orbital part | -0.79 | Fusiform gyrus | -0.91 |
| Rolandic operculum | -1.37 | Postcentral gyrus | -1.26 |
| Supplementary motor area | -1.53 | Superior parietal gyrus | -0.94 |
| Olfactory cortex | -0.63 | Supramarginal gyrus | -1.10 |
| Superior frontal gyrus, medial orbital | -0.76 | Angular gyrus | -0.94 |
| Gyrus rectus | -1.27 | Precuneus | -0.62 |
| Insula | -0.94 | Paracentral lobule | -0.69 |
| Median cingulate and paracingulate gyri | -1.12 | Lenticular nucleus, putamen | -0.69 |
| Posterior cingulate gyrus | 0.42 | Heschl gyrus | -1.26 |
| Hippocampus | -0.45 | Superior temporal gyrus | -1.24 |
| Parahippocampal gyrus | -0.64 | Temporal pole: superior temporal gyrus | -1.40 |
| Amygdala | -0.72 | Middle temporal gyrus | -0.98 |
| Calcarine fissure and surrounding cortex | -0.81 | Temporal pole: middle temporal gyrus | -0.74 |
| Lingual gyrus | -1.24 | Inferior temporal gyrus | -0.79 |
| Superior occipital gyrus | -0.80 |  |  |
| Middle occipital gyrus | -0.95 | **Betweenness Centrality** |  |
| Fusiform gyrus | -1.39 | Rolandic operculum | -0.35 |
| Superior parietal gyrus | -0.61 | Inferior temporal gyrus | -1.35 |
| Inferior parietal, but supramarginal and angular gyri | -0.89 |  |  |
| Supramarginal gyrus | -1.07 |  |  |
| Angular gyrus | -0.66 |  |  |
| Paracentral lobule | -0.68 |  |  |
| Heschl gyrus | -0.88 |  |  |
| Superior temporal gyrus | -1.29 |  |  |
| Temporal pole: superior temporal gyrus | -1.36 |  |  |
| Middle temporal gyrus | -1.19 |  |  |
| Temporal pole: middle temporal gyrus | -0.85 |  |  |
| Inferior temporal gyrus | -1.94 |  |  |

*E_local_* = local efficiency, *E_nodal_* = nodal efficiency.
